# Optimizing Adrenaline Administration in Anaphylaxis: Clinical Practice Considerations and Safety Insights

**DOI:** 10.1002/clt2.70085

**Published:** 2025-08-06

**Authors:** Motohiro Ebisawa, Antonella Muraro, Margitta Worm, Constance H. Katelaris, Guillaume Pouessel, Johannes Ring, George Du Toit, Adam T. Fox

**Affiliations:** ^1^ Clinical Research Center for Allergy and Rheumatology NHO Sagamihara National Hospital Sagamihara Kanagawa Japan; ^2^ Food Allergy Referral Centre Department of Woman and Child Health Padua University Hospital Padua Italy; ^3^ Division of Allergy and Immunology Department of Dermatology, Venerology, and Allergy Campus Charité Mitte Universitätsmedizin Berlin Berlin Germany; ^4^ Immunology and Allergy Unit Department of Medicine Campbelltown Hospital and Western Sydney University Sydney New South Wales Australia; ^5^ CHU Lille, Pediatric Pulmonology and Allergy Department Hôpital Jeanne de Flandre Lille France; ^6^ Department of Dermatology Allergology Biederstein Technical University Munich (TUM) Munich Germany; ^7^ King's College London School of Life Course Sciences and School of Immunology & Microbial Sciences London UK; ^8^ Children’s Allergy Service Evelina London Children's Hospital London UK; ^9^ Guy's and St Thomas’ NHS Foundation Trust London UK

**Keywords:** adrenaline, anaphylaxis, autoinjector, life‐threatening, safety

## Abstract

**Background:**

Anaphylaxis is an acute, severe, and potentially fatal reaction marked by the fast onset of symptoms and organ involvement that may lead to death from vascular collapse or airway obstruction. Despite adrenaline (epinephrine) being the first‐line medication for reversing anaphylactic symptoms, misconceptions about its safe and correct use persist and lead to improper administration.

**Methods:**

This review provides a comprehensive overview of clinical use of adrenaline autoinjectors (AAIs) in the management of anaphylaxis, key safety considerations, and pharmacokinetic/pharmacodynamic profile of three of the currently marketed AAIs.

**Results:**

When administered intramuscularly (IM) at the recommended dose for anaphylaxis, adrenaline is considered safe; however, adequate training in emergency care is essential to minimize dosage errors and mitigate safety risks. In specific situations, such as refractory anaphylaxis, intravenous (IV) administration is advised under specialized settings due to the potential risk of severe cardiovascular complications that can result from dosing errors.

**Conclusion:**

Although adrenaline can cause mild and transient side effects even when administered correctly at the recommended dosage, the potential side effects should not deter its use in critical situations such as anaphylaxis. This review aims to highlight the role of AAIs in improving patient outcomes during anaphylactic emergencies.

## 1 Introduction

Anaphylaxis is an acute, potentially severe and fatal, systemic hypersensitivity reaction characterized by the rapid onset of symptoms and signs that may cause death by airway obstruction or vascular collapse [1]. The most common triggers of anaphylaxis are medications, foods (particularly peanut, tree nuts, shellfish, milks, and egg), and insect venoms [2, 3, 4]. Signs and symptoms usually develop within a few seconds to 30 min, depending on the route of exposure, but there may rarely be a quiescent period of 1–8 h before the development of a second reaction (a biphasic response of the affected individuals) [2]. The most common clinical cutaneous manifestations include angioedema, urticaria, erythema, and pruritus; however, skin signs are absent in 10%–30% of anaphylaxis reactions [5].

Anaphylaxis is an increasingly prevalent problem worldwide. The prevalence of anaphylaxis in the United States is between 1.6% and 5.1%. Further, 1 in 20 individuals in the United States are said to have experienced anaphylactic reactions, with 225 deaths per year attributed to anaphylaxis [6]. In Europe, the incidence rate of anaphylaxis has been shown to be 1.5–7.9 per 100,000 person/year [7]. Anaphylactic reactions require emergency medical treatment due to serious reactions and subsequently this may have a major impact on individuals' quality of life (QoL) leading to psychological issues, particularly anxiety and fear [7]. Diagnosis and management of anaphylaxis are challenging since allergic reactions are most often immediate, unexpected, with an unpredictable time course, and with varying severity of clinical presentations [8]. Therefore, prompt recognition and management of anaphylaxis is imperative; however, it is often under‐recognized and treated inadequately, in all age groups, particularly in infants [9]. Adrenaline (epinephrine) is the drug of choice for the treatment of anaphylaxis and should be administered promptly in the event of an anaphylactic reaction. Delaying adrenaline administration in patients experiencing anaphylaxis has been associated with poor outcomes and an increase in the occurrence of biphasic reactions [10, 11].

The international and national guidelines for anaphylaxis recommend prompt intramuscular (IM) injection of adrenaline into mid‐anterolateral (AL) thigh as the first‐line treatment [1, 12, 13]. Despite this, the European Anaphylaxis Register shows that only a small percentage of anaphylactic events are managed with adrenaline, highlighting notable gaps in the implementation of these established guidelines [14]. Recent approval of the adrenaline nasal spray is expected to provide an alternative to existing AAIs for some patients [15]. In addition to the management of anaphylaxis through adrenaline administration, the European Academy of Allergy and Clinical Immunology (EAACI) guidelines also recommend removing the trigger, calling for help/assistance, and correcting the positioning of the patient. Other second‐line interventions that are recommended include high‐flow oxygen, intravenous (IV) fluids, inhaled short‐acting bronchodilators, and nebulized adrenaline (if stridor, as supplement to intramuscular [IM] adrenaline) for the management of anaphylaxis [16, 17].

Third‐line interventions include antihistamines and corticosteroids [16]. Antihistamines have some efficacy in managing cutaneous symptoms and may also be effective as adjunct therapy for urticaria and angioedema; however, they do not promptly address the serious manifestations that can be fatal, such as upper airway obstruction, bronchospasm, hypotension, and the cardiovascular clinical manifestations, that is sudden drop in the blood pressure. Furthermore, antihistamines and corticosteroids have a prolonged time to onset of action (0.7–3 h) and time to peak plasma concentration (0.8–2.8 h) can take several hours; therefore, these medications are not adequate as first‐line treatment for anaphylaxis [3]. Glucocorticoids are frequently administered in anaphylaxis due to their ability to prevent extended symptoms and avert biphasic reactions. However, the evidence supporting their effectiveness is limited, and there may be potential risks associated with their use in pediatric patients [17].

Several studies have assessed the efficacy of AAIs by evaluating injection speed, injection depth, and drug dose [18]. However, data on the safety profile and usage of adrenaline, particularly regarding its delivery via adrenaline auto‐injectors (AAIs), is limited. The objective of this review is to provide updated insights on the clinical use of adrenaline in anaphylaxis management, including current guideline recommendations, practical considerations for adrenaline administration through AAIs, and potential safety concerns related to their use. Furthermore, we also provide some suggestions on how physicians and HCPs can educate and counsel individuals who are at risk of anaphylaxis, thereby empowering them to self‐administer AAIs during emergency situations and reducing the fear surrounding their use in the context of anaphylaxis management.

## 2 Literature Search

An unstructured literature search was conducted in the PubMed, Cochrane library, Google scholar, ScienceDirect and ResearchGate databases using the following keywords: “adrenaline”, “adrenaline autoinjectors”, “anaphylaxis”, “prevalence”, “anaphylaxis management”. To identify articles specifically addressing potential safety concerns with the use of AAIs, additional keywords such as “side effects” and “serious adverse events” were used. Additionally, websites related to international guidelines for treatment recommendations were also searched. No date restrictions or geographical limitations were followed in the literature search. Only articles in English language and human studies were included in the literature search.

## 3 Adrenaline: First‐Line Treatment for Anaphylaxis

**FIGURE 1:**
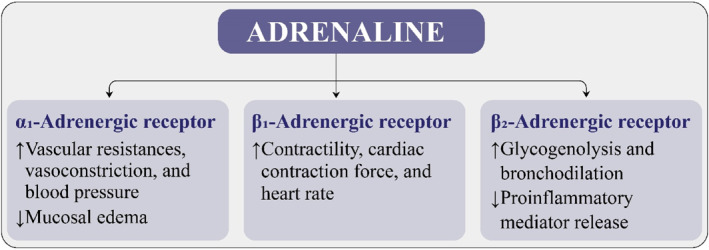
Pharmacological effects of adrenaline in the treatment of anaphylaxis.

Adrenaline has a narrow therapeutic index and has bidirectional actions that are concentration dependent. The life‐saving pharmacologic effects of adrenaline, such as vasoconstriction, reduction in mucosal edema, bronchodilation, and reduction in release of histamine, tryptase, and other proinflammatory mediators (Figure 1) cannot be separated from pharmacologic side effects like pallor, anxiety, tremor, and palpitations [19, 20].

The unpredictable nature of anaphylaxis, as well as its rapid progression once initiated, necessitates the availability of self‐administered emergency treatment [8]. The prompt administration of adrenaline is facilitated by AAIs, thereby significantly improving treatment outcomes in individuals experiencing anaphylaxis [3, 21]. Consequently, it is imperative for patients who are at risk of anaphylaxis to carry two AAIs to ensure immediate and adequate treatment [22]. The rationale for prescribing two AAIs in each emergency kit is based on the following factors: around 10% patients may need a second dose of adrenaline due to an inadequate response to the first dose; there is a possibility of an AAI malfunction or incorrect injection; and there may be delays in receiving medical assistance [17].

Several guidelines outline specific recommendations for the appropriate doses of IM adrenaline through AAIs in the management of anaphylaxis, ensuring that treatment is both effective and timely. The EAACI guidelines recommend prescribing 0.15 mg adrenaline for children weighing 7.5 kg to 25–30 kg, 0.3 mg adrenaline for children weighing 25–30 kg, and at least 0.3 mg adrenaline for adolescents and adults at risk of anaphylaxis [16]. As practical consideration, for adolescents and adult patients, a 0.3 mg device is recommended although a higher dose of 0.5 mg device can be considered when a patient is overweight or has experienced a previous episode of life‐threatening anaphylaxis [17]. The guidelines of the Australasian Society of Clinical Immunology and Allergy (ASCIA) recommend 0.15 mg adrenaline for children weighing 7.5–20 kg and ≤ 5 years of age; 0.3 mg adrenaline for children weighing > 20 kg and ≥ 5 years of age; and 0.5 mg adrenaline for those weighing > 50 kg and ≥ 12 years of age [23]. Another set of guidelines from the Resuscitation Council UK have similar recommendation regarding adrenaline dose: 0.1–0.15 mg for children < 6 months; 0.15 mg for children aged 6 months–6 years; 0.3 mg for children between 6 and 12 years of age; and 0.5 mg for children and adults over the age of 12 years [24]. The dosage‐related details of the currently marketed AAIs are presented in Table 1.

**TABLE 1:** List of currently marketed adrenaline auto‐injectors [25].

Properties	EpiPen	Jext	AUVI‐Q	Anapen	Emerade
Dosage and body weight	150 μg/0.3 mL for children weighing 7.5–25 kg and 300 μg/0.3 mL for children and adults weighing > 30 kg	150 μg/0.3 mL in children weighing 15–30 kg and 300 μg/0.3 mL in children and adults weighing > 30 kg	0.1 mg/0.1 mL for patients weighing 7.5–15 kg 0.15 mg/0.15 mL for patients weighing 15–30 kg 0.3 mg/0.3 mL for patients weighing ≥ 30 kg	150 μg/0.3 mL in children weighing 15–30 kg 300 μg/0.3 mL in children, adolescents, and adults weighing > 30 kg 500 μg/0.3 mL in adolescents and adults weighing > 60 kg	

Studies have shown that IM adrenaline administration by AAIs is substantially better at delivering a therapeutically relevant dose with significantly higher plasma adrenaline concentrations than is achieved with a normal syringe injection [10, 26]. A prospective controlled study demonstrated that the low‐cost, “low‐tech” adrenaline ampule/syringe/needle technique is impractical for parents of infants experiencing anaphylaxis, as they struggled to draw the correct dose quickly and accurately [27]. Further, while most of the HCPs, who also participated in this study, were able to draw the dose promptly, there were inconsistencies in adrenaline concentrations in the ampules and challenges in measuring low volumes (< 0.1 mL) of adrenaline [27]. This reinforces the preference for IM use of AAIs as a more effective method for anaphylaxis management. The IM route is also recommended even in healthcare settings like hospital, except for perioperative anaphylaxis, for which the IV route is highly recommended [17, 28, 29]. Moreover, IV adrenaline may be necessary for treating refractory anaphylaxis in a clinical setting, where it is administered under careful supervision of skilled HCPs who monitor its effects [30, 31]. This is vital as the rapid IV administration of improperly high doses may lead to serious adverse events (AEs), such as myocardial ischemia, dysrhythmias, and pulmonary edema [21]. This risk is particularly pronounced in older individuals [32, 33]. The potential safety issues, particularly cardiovascular complications associated with the administration of adrenaline, are elaborated upon in a separate section.

## 4 Clinical Safety and Practical Recommendations for Adrenaline Administration Through AAIs

Adrenaline is considered as a safe medication for the treatment of anaphylaxis when administered as per the recommended dose and route [34, 35]. Intramuscular adrenaline (1 mg/mL) should be given at a rate of 0.01 mL per kilogram of body weight up to a total of 0.5 mg per dose and can be repeatedly given in short intervals (5–10 min) until the patient's condition is stabilized [36]. However, recent guidelines from the Resuscitation Council of UK have proposed that IV adrenaline infusion should be given in patients with refractory anaphylaxis defined as a reaction requiring ongoing treatment despite two (appropriate) doses of IM adrenaline [30, 37]. An observational study in 58 adults treated with adrenaline for anaphylaxis reported that the side effects of IM adrenaline, which were described in fewer than one in five patients during anaphylaxis treatment, were usually mild and transient, such as tremors, palpitations, and anxiety. Additionally, none of the AEs were due to the use AAI [38]. Therefore, limiting adrenaline administration owing to anticipated side effects in an emergency circumstance like anaphylaxis would not be warranted. The benefits of administering appropriate dose and choosing the IM route substantially outweighs the risks [39].

Two separate studies from France have documented AEs associated with AAIs, which were reported to the 30 pharmacovigilance centers and the network of the 8 poison control centers [40, 41]. The survey conducted from 2018 to 2022 among poison control centers identified 315 AAI‐related AEs, most of which were mild in the context of accidental injection in the finger [41]. Systemic symptoms have been reported in 22 (7.0%) cases, but none were considered as serious: tachycardia (*n* = 20, 6.3%), hypertension (*n* = 3, 1.0%), dizziness (*n* = 2, 0.6%), tremors (*n* = 2, 0.6%), vomiting and chest pain (*n* = 1, 0.3%, each).

## 5 Potential Safety Concerns Related to Adrenaline and AAIs

### 5.1 Cardiovascular (CV) Complications

Previous studies have reported that IV administration of adrenaline for anaphylaxis may result in severe CV complications [18, 42]. The majority of CV AEs associated with IV adrenaline treatment have been shown to have occurred due to adrenaline overdosage [18, 35, 43]. Adrenaline enhances cardiac output by acting on the β1‐adrenergic receptors and promotes vasoconstriction via activation of the α1‐adrenergic receptor, which might result in myocardial ischemia, ventricular tachycardia, and myocardial infarction [44, 45]. An observational cohort study compared the rates of CV AEs and overdoses when adrenaline was administered through different routes in emergency departments to patients with anaphylaxis. The results showed that 10% of patients with anaphylaxis who received adrenaline as an IV bolus experienced CV AEs compared to only 1.3% patients who received IM adrenaline (*p* = 0.006) [42]. Further, adrenaline overdosage was only noted when it was administered as an IV bolus [42]. This shows that even in an emergency setting, there is limited awareness on appropriate usage and route of administration of adrenaline [3, 25]. Education and training on usage aspects may help reduce CV complications and overdoses when using adrenaline to treat anaphylaxis [17, 46]. Access to AAIs in emergency situations, especially within medical service vehicles, may contribute to a decrease in CV AEs and ensure timely administration of IM adrenaline, in accordance with relevant guidelines.

### 5.2 Risk of Overdosing and Needle Injuries

Adrenaline overdosing may occur due to misunderstanding of the IM dose recommended for the treatment of anaphylaxis (0.01 mL per kilogram of body weight up to a total dose of 0.5 mg per dose of 1 mg/mL concentration [1:1000] from childhood to adulthood) and the IV dose used for anaphylactic shock treatment (in children, 1 μg/kg and in adults 0.1 mg of 0.1 mg/mL concentration [1:10,000]), and the IV dose for cardiac arrest (in adults, 1 mg of 0.1 mg/mL concentration [1:10,000]) [47]. Only 61% of physicians were able to accurately calculate the volume of adrenaline required, when provided with the concentration and intended mass, indicating that miscalculations may lead to overdose and fatal AEs [43, 48]. Overdosage of adrenaline may result in serious AEs such as elevated arterial pressure, cerebrovascular hemorrhage, pulmonary edema, cardiac arrhythmias, extreme pallor and coldness of the skin, metabolic acidosis, and kidney failure, necessitating prompt corrective actions [49]. Stress cardiomyopathy, also known as Takotsubo syndrome, is also reported in patients receiving adrenaline in anaphylaxis. It is a type of non‐ischemic cardiomyopathy characterized by an acute and reversible dysfunction of the left ventricle with typical apical ballooning, usually with subsequent complete recovery that may be associated with using exogenous catecholamine administration, including adrenaline IM or IV in the context of anaphylaxis [50].

AAIs are designed to be a user‐friendly method for delivering a precise amount of IM adrenaline and for consistently releasing enough adrenaline to the systemic circulation [51]. Moreover, injection site, biphasic adrenaline levels, and cardiovascular responses are important factors to increase the AAIs' efficacy. Latest pharmacokinetic (PK) studies have shown that the use of optimal needle length is essential for effective administration of adrenaline [25, 52]. In order to reduce needle stick injuries, adequate training and education regarding correct use should be given to patients and caregivers.

The most common injuries reported with AAIs are unintentional injections. In the French network of 8 poisons control centers, 93% of the 315 adverse effects related to accidental injection using AAI occurred due to an accidental use not related to an allergic reaction and were mild [41]. Local symptoms were reported in 187 cases (59.4%): pain (*n* = 136, 43.2%), pallor (*n* = 96, 30.5%), coldness (*n* = 66, 21.0%) [41]. Lacerations, embedded needles, needle hooking injuries from AAIs are less common, but pose a risk, especially in children. Accidental digital injections while using AAIs are also reported and can be associated with profound ischemia of the affected digit [53]. Administering phentolamine directly into the site of injury has been shown to be the most effective strategy for reversing the symptoms and treating ischemic digits. A treatment algorithm for the management of these injuries has also been proposed [54].

Reports indicate that administering adrenaline via an AAI into the thighs of uncooperative children, who may kick or move during the injection, has resulted in instances of lacerations, bent needles, and embedded needles [53]. To minimize these injections‐related injuries, the needle injection time has to be reduced and caregivers/physicians should be instructed to hold the child's leg firmly in place and limit movement prior to and during injection. Further, the reporting of device failures can result in improvements in device design and performance, which could subsequently reduce the occurrence of AEs [53].

### 5.3 Storage Conditions and Expired Adrenaline Formulations

Adrenaline is an unstable chemical with a relatively short shelf‐life and is recommended to be stored at 20°C–25°C (68°F–77°F). It is essential that AAIs be replaced prior to their expiration date to ensure proper management of anaphylaxis. Additionally, HCPs should strongly communicate to patients the critical need to refill any AAIs that have expired [55].

A systematic literature review investigating the effect of excessive heat or cold on adrenaline showed that short‐term exposure to extreme temperature or real‐world temperature fluctuations did not have an effect on adrenaline degradation [56]. Another study carried out with two of the currently marketed AAI brands (Epipen and Jext) using 12 devices of each device demonstrated no significant loss in the amount of adrenaline solution ejected after multiple freeze‐thaw cycles (freezing at −80°C and then allowing it to return to room temperature); however, further investigations are needed to assess the safety and efficacy of AAIs after exposure to very low temperature [57]. Other studies have shown that most devices retained at least 90% of the labeled concentrations for up to 24 months past the designated expiry date, which was within the limits (90%–115%) of US Pharmacopeia standards and US Food and Drug Administration (US FDA) [58]. However, other compounds of the formulation, which are necessary for stability of the product, were not assessed in these studies and therefore its use beyond expiration date is not recommended by US FDA, European Medicines Agency (EMA), and other authorities [59, 60].

## 6 Pharmacokinetics/Pharmacodynamics (PK/PD) Profile of AAIs

The TEN study by Baker et al., is the first study to investigate time required by an AAI to deliver adrenaline into muscle. The study reported that 95.9% of adrenaline is absorbed into muscle tissue in 1 s, and that holding the device in position for 1 s yields results comparable to those achieved after 10 s [61]. In this section, we briefly discuss the PK/PD profile of three of the AAIs that are currently marketed, namely EpiPen, Emerade, and Anapen.

### 6.1 EpiPen PK/PD Data [21, 62]:

When comparing EpiPen versus IM syringe, an adrenaline injection (300 μg dosage) with EpiPen led to a larger *C*_max_ and a shorter *T*_max_, suggesting a fast absorption through low‐ to high skin‐to‐muscle distance (STMD). This was noted despite EpiPen’s needle being shorter than the IM syringe’s (16 mm vs. 12–40 mm). *T*_max_ was delayed for participants in the EpiPen group who had higher STMD compared to those who had low and moderate STMD, indicating a delayed absorption in the obese population (30 vs. 9 vs. 10.5 min, respectively). Overall, *C*_max_ was higher in women compared to men in all STMD categories (*C*_max_ range: 520–640 vs. 400–480 pg/mL, respectively). In low‐to‐high STMD groups, early systemic adrenaline exposure (measured as partial AUC‐time [pAUC] curve from 0 to 30 min or AUC 0–30) was higher with EpiPen 300 μg than with IM syringe 300 μg. The pAUC values estimated at 6 and 15 min were higher with EpiPen 300 μg compared to IM syringe, respectively, indicating an early adrenaline exposure.

### 6.2 Emerade PK/PD Data [63]:

A lower *C*_max_ was observed with Emerade 300 and 500 μg compared to EpiPen 300 and Jext 300 μg within the first 5 minutes post injection in a study evaluating PK/PD of normal weight and obese participants with low‐to‐high STMDs (98.25 vs. 145.4 vs. 210.7 vs. 150.2 pg/mL, respectively). This finding suggests that delayed *T*_max_ was seen with both dosages of Emerade (300 and 500 μg) compared to EpiPen (300 μg), which is consistent with these findings. Contrary to EpiPen and Jext, Emerade has a longer needle (23 vs. 16 vs. 15 mm). With Emerade 500 μg, a greater *C*_max_ was seen after 40–50 min. Emerade 500 μg and Emerade 300 μg had different intra‐dosing *C*_max_ ranges (220.2–298.5 pg/mL and 341.5–543.4 pg/mL, respectively). These results imply that Emerade, in comparison to EpiPen and Jext, requires larger dose to attain higher *C*_max_. Overall, the Emerade PK/PD data revealed considerable variability and a delayed *T*_max_, particularly in persons with high STMD, which may have indicated a delayed start to action and less than ideal effects in this population.

### 6.3 Anapen PK/PD Data [35, 62]:

In both low‐to‐high STMD individuals (10–15 mm), Anapen 300 μg lead to a higher *C*_max_ with a higher systemic adrenaline exposure (measured as AUC, AUC_0–20_, i.e., time curve from 0 to 20 min), indicating faster and greater adrenaline absorption than IM syringe 300 μg. Despite having a shorter needle length than an IM syringe, this was the case (10.5 vs. 25.4 mm). It is interesting to note that in high STMD individuals, the depth of the adrenaline depot was approximately 11 mm, which is nearly the same as the 10.5 mm Anapen needle length (though adrenaline depot depths have not been calculated in the other two studies). This shows that SC injections were administered to obese individuals with high STMD (15 mm) as opposed to IM. Anapen 300 μg achieved high *C*_max_ in patients with high STMD. Additionally, in those with low STMD, Anapen 300 μg showed a lower *C*_max_ compared to IM syringe 500 μg (377 vs. 401.2 pg/mL, respectively), whereas the comparison with IM syringe 500 μg was not possible in patients with high STMD. In those with medium STMD, PD parameters such HR alterations were more pronounced with Anapen 300 μg compared to IM syringe 300 and 500 μg. Additionally, Anapen 500 μg PK/PD profiling was not done in this investigation.

## 7 Discussion

This review highlights the central role of adrenaline in anaphylaxis management, including recommended administration strategies, PK/PD profile of AAIs, and safety considerations. The evidence from published studies shows that adrenaline is vital in the management of anaphylaxis. There are reports of mild AEs such as transient pallor, tremor, anxiety, and palpitations, and these effects are inextricably linked to adrenaline’s positive benefits [64]. Maximum benefit and safety are attained when recommended doses and IM route of administration of adrenaline is used. IV adrenaline is indicated in the context of perioperative setting, in case of a severe cardiovascular compromise (including cardiac arrest), or if anaphylaxis is refractory to the first IM adrenaline doses [37, 65].

The AAIs are currently the recommended mode of adrenaline delivery in emergency situations except in the perioperative settings; however, other needle‐free alternatives that have been recently approved or are currently under development may serve as additional options for the management of anaphylaxis [15, 66]. Currently, there is widespread consensus that the benefits of administering an AAI promptly to treat anaphylaxis outweighs the associated risks [25, 42]. As per the EAACI recommendations, prescribing and carrying two AAIs is preferred, as approximately 10% patients may need a second dose of adrenaline if the first dose does not yield sufficient response. Further, majority of the patients respond to one dose, while any additional doses are typically administered by emergency services [16]. Experts tend to prescribe AAIs more often in specific circumstances (e.g., mastocytosis, asthma, excess body weight, a history of severe anaphylaxis, or limited access to rapid emergency), which is in line with the guidelines. There is a lack of understanding and awareness among physicians prescribing AAIs, which needs to be addressed immediately for better anaphylactic treatment [67]. Patients with the below listed conditions need to carry two AAIs with correct dosage as per their body weight [17]:

- Co‐existing unstable or moderate to severe, persistent asthma, and food allergy
- Co‐existing mast cell diseases and/or elevated baseline tryptase concentration
- Lack of rapid access to medical assistance to manage an episode of anaphylaxis due to geographical or language barriers
- Previous requirement for more than one dose of adrenaline prior to reaching hospital

Counseling and training, such as educational programs and seminars are essential to create awareness among physicians and the public regarding safety and efficacy of AAIs. Moreover, physicians should educate patients on how to use the device properly and monitor them frequently to minimize any errors [68]. Clinicians and policymakers must collaborate to raise anaphylaxis awareness and make AAIs available to all patients with a history of anaphylaxis or those who are at risk of anaphylaxis. In addition, we must keep in mind that the availability of AAIs for use in the first‐aid treatment of anaphylaxis is limited to only 32% of all 195 world countries [69]. It is essential for physicians, lawmakers, patients, and pharmaceutical companies to collaborate in order to enhance awareness and advocate for the global accessibility of AAIs, which are vital for the effective management of anaphylaxis and the prevention of avoidable morbidity and mortality.

## 8 Conclusion

This review article demonstrates that IM adrenaline is a safe option when given at an adequate dose in the context of anaphylaxis contrary to IV adrenaline which exposes individuals to CV AEs and should only be given by experienced specialists in specific situations such as perioperative or refractory anaphylaxis. For rapid onset of action, AAIs play a vital role in self‐administering adrenaline. While adrenaline may induce mild and temporary side effects when administered appropriately at the recommended dosage, these potential effects should not discourage its application in life‐threatening situations such as anaphylaxis.

## Author Contributions

**Motohiro Ebisawa:** conceptualization, supervision, project administration, writing – original draft, writing – review and editing, validation, formal analysis, investigation. **Antonella Muraro:** investigation, validation, writing – original draft, writing – review and editing. **Margitta Worm:** investigation, validation, writing – original draft, writing – review and editing. **Constance H. Katelaris:** investigation, validation, writing – original draft, writing – review and editing. **Guillaume Pouessel:** investigation, validation, writing – original draft, writing – review and editing. **Johannes Ring:** investigation, validation, writing – original draft, writing – review and editing. **George Du Toit:** investigation, validation, writing – original draft, writing – review and editing. **Adam T. Fox:** investigation, validation, writing – original draft, writing – review and editing.

## Conflicts of Interest

The authors declare no conflicts of interest.

## Data Availability

Data sharing is not applicable to this article as no new data were created or analyzed in this study.
